# Genomic alterations in neuroendocrine cancers of the ovary

**DOI:** 10.1186/s13048-016-0259-2

**Published:** 2016-08-26

**Authors:** George Yaghmour, Philippe Prouet, Eric Wiedower, Omer Hassan Jamy, Rebecca Feldman, Jason C Chandler, Manjari Pandey, Mike G Martin

**Affiliations:** 1 The West Cancer Center, 1588 Union Ave., Memphis, TN 38104 USA; 2 Department of Hematology & Oncology, The University of Tennessee Health Science Center, 956 Court Ave., Suite H310A, Memphis, TN 38163 USA; 3 Department of Internal Medicine, The University of Tennessee Health Science Center, 956 Court Ave., Suite H314, Memphis, TN 38163 USA; 4 Caris Life Sciences, 4750 S. 44th Place, Phoenix, AZ 85040 USA

**Keywords:** Gynecologic malignancies, Small cell carcinoma of ovary, Genomic profiling, Actionable mutation, Chemotherapy

## Abstract

**Background:**

As we have previously reported, small cell carcinoma of the ovary (SCCO) is a rare, aggressive form of ovarian cancer associated with poor outcomes. In an effort to identify new treatment options, we utilized comprehensive genomic profiling to assess the potential for novel therapies in SCCO.

**Methods:**

Patients with SCCO, SCCO-HT (hypercalcemic type), neuroendocrine tumors of the ovary (NET-O), and small cell carcinoma of the lung (SCLC) profiled by Caris Life Sciences between 2007–2015 were identified. Tumors were assessed with up to 21 IHC stains, in situ hybridization of cMET, EGFR, HER2 and PIK3CA, and next-generation sequencing (NGS) as well as Sanger sequencing of selected genes.

**Results:**

Forty-six patients with SCCO (10 SCCO, 18 SCCO-HT, 18 NET-O) were identified as well as 58 patients with SCLC for comparison. Patients with SCCO and SCCO-HT were younger (median 42 years [range 12–75] and 26 years [range 8–40], respectively) than patients with NET-O 62 [range 13–76] or SCLC 66 [range 36–86]. SCCO patients were more likely to be metastatic (70 %) than SCCO-HT (50 %) or NET-O (33 %) patients, but at a similar rate to SCLC patients (65 %). PD1 expression varied across tumor type with SCCO (100 %), SCCO-HT (60 %), NET-O (33 %) vs SCLC (42 %). PDL1 expression also varied with SCCO (50 %), SCCO-HT (20 %), NET-O (33 %) and SCLC (0 %). No amplifications were identified in cMET, EGFR, or HER2 and only 1 was found in PIK3CA (NET-O). Actionable mutations were rare with 1 patient with SCCO having a BRCA2 mutation and 1 patient with NET-O having a PIK3CA mutation. No other actionable mutations were identified.

**Conclusions:**

No recurrent actionable mutations or rearrangements were identified using this platform in SCCO. IHC patterns may help guide the use of chemotherapy in these rare tumors.

## Background

Small cell carcinoma of the ovary (SCCO) is a very rare and aggressive form of ovarian cancer that carries a poor prognosis despite early median age at diagnosis. SCCO presents challenges for diagnosis, prediction of outcomes, and overall treatment strategies [1, 2]. Due to limited data, there are no defined treatment protocols [3].

Molecular mechanisms underlying development and progression of SCCO remain poorly understood. Inactivation in SMARCA4, which codes for BRG1, in small cell carcinoma of the ovary hypercalcemic type (SCCO-HT) has been described in 75-100 % of cases. SMARCA4 is a member of the SWI/SNF chromatin-remodeling gene complex, which has been shown to be mutated in several different cancers. SMARCA4 inactivation appears to be a specific diagnostic marker in SCCOHT [4–7]. This inactivation, however, does not define any treatment strategies that would be defined with actionable abnormalities.

Profiling of rare tumors using the Caris Molecular Intelligence platform has yielded potential targets in various tumor types. Burzawa, et al. analyzed on 78 samples of small cell cancer of the cervix using Caris Molecular Intelligence (53 samples) and a 50-gene next-generation sequencing (NGS) platform (25 samples). They showed elevated expression of TOPO2A and TOPO1 implying sensitivity to etoposide and topotecan, respectively. They also described potentially targetable mutations in *Akt1*, *KRAS*, *PIK3CA*, and *TP53*. One of the patients who had a *KRAS* mutation showed a complete response to MEK inhibitor therapy ongoing for more than 12 months [8]. PIK3CA mutations were identified as potential targets in small call carcinoma of the breast in another study using the Caris Molecular Intelligence platform [9].

Caris Molecular Intelligence is a multi-platform tumor profiling service that includes gene sequencing (next-generation sequencing [NGS] and Sanger), protein expression analysis (immunohistochemistry [IHC]), and gene copy number and translocation analysis (chromogenic or fluorescence in situ hybridization [CISH or FISH]). As patients living with these malignancies have few therapeutic options, we hypothesized that Caris Molecular Intelligence may provide clinically relevant information for women with SCCO [10].

## Methods

After obtaining Institutional Review Board approval from the University of Tennessee we queried the Caris Life Sciences database for patients profiled by Caris Molecular Intelligence using the keywords “small cell” to search in the clinical history and diagnosis fields for cases of SCCO, SCCO-HT (hypercalcemic type), neuroendocrine tumors of the ovary (NET-O) from 2007–2015. Comparative data was pulled for SCLC from April 2015- September 2015. Caris registry is a proprietary database to which the investigators have access for research purposes, which collates and categorizes molecular alterations and cancer types across patients who have had cancer molecular testing done throughout The United States using a multi-platform tumor profiling service that includes gene sequencing with NGS and Sanger, protein expression analysis with IHC, and gene copy number and translocation analysis with CISH or FISH.

Clinicopathologic characteristics of patients with SCCO, SCCO-HT, NET-O and SCLC patients were identified, including age and whether metastatic disease was present at the time of profiling. IHC analysis was performed using commercially available detection kits and automated staining techniques (Benchmark XT, Ventana, Tucson, AZ; and AutostainerLink 48, Dako, Carpinteria, CA). Tumors were assessed with up to 25 IHC stains (ALK, AR, BCRP, c-KIT, ER, PR, cMET, EGFR, HER2, IGF1R, PTEN, PD-1, PDGFR, PD-L1, ERCC1, TS, MGMT, RRM1, TLE3, TUBB3, SPARC, TOP2A, TOPO1, MRP1, PGP). Gene copy number alterations of cMET, EGFR, HER2, PIK3CA, and TOP2A were analyzed by DNA ISH using (FISH and/or CISH probes as part of automated staining techniques (Benchmark XT, Ventana, Tucson, AZ) and automated imaging systems (BioView, Billerica, MA). The ratio of gene to pericentromeric regions of chromosome 7 (EGFR, cMET), 17 (HER2, TOP2A) and 3 (PIK3CA) were used to determine increases in gene copy number. Tumors also underwent NGS analysis, which is a form of parallel sequencing that greatly enhances the efficiency of identifying both somatic and germline mutations [11]. NGS sequencing was performed on genomic DNA isolated from tumor tissue using the Illumina MiSeq platform. PCR products were bi-directionally sequenced using the BigDye Terminator v1.1 chemistry, analyzed using the 3730 DNA Analyzer (Applied Biosystems, Grand Island, NY). Sequence traces were analyzed using Mutation Surveyor software v3.25 (Soft Genetics, State College, PA). NGS and Sanger sequencing of a 47-gene panel (ABL, AKT, ALK, APC, ATM, BRAF, BRCA1, BRCA2, CDH1, cKIT, cMET, CSF1R, CTNNB1, EGFR, ERBB4, FBXW7, FGFR1, FGFR2, FLT3, GNA11, GNAQ, GNAS, HER2, HNF1A, HRAS, IDH1, JAK2, JAK3, KDR, KRAS, MLH1, MPL, NOTCH1, NPM1, NRAS, PDGFRA, PIK3CA, PTEN, PTPN11, RB, RET, SMAD4, SMARCB1, SMO, STK11, TP53, VHL) was evaluated for somatic mutations. Coding changes were accessed for pathogenicity using PolyPhen-2 [10, 12].

Retrospective analysis of biomarker frequency distributions was attained using standard descriptive statistics evaluating the incidence of the aforementioned genomic alterations in these tumors by Caris Molecular Intelligence profiling. The two-tail Fisher’s exact test analyzed whether frequencies differed by subgroup, specifically to determine if any non-random associations between SCCO and SCLC existed. A *p*-value <0.05 was considered statistically significant and all *p*-values were 2-sided.

## Results

Forty-six unique patients with SCCO were identified that had profiling between 2007–2015. These included 10 patients with SCCO with median age of 42 (range 12–75), 18 patients with “small cell/hypercalcemic” (SCCO-HT) with median age of 26 (range 8–40), and 18 patients with “neuroendocrine” (NET-O) with median age of 62 (range 13–76). 70 % of SCCO patients, 50 % of SCCO-HT patients, and 33 % NET-O patients had metastatic disease at presentation. For comparison, 58 patients with SCLC were identified. The median age was 66 (range 36–86), and 65 % had metastatic disease at presentation (Table 1).

**Table 1:** Median age and % Metastatic disease at presentation of SCCO, SCCO-HT, NET-O, and SCLC

Small Cell Cancer Type	Median Age	%Metastatic
SCCO (*n* = 10)	42 (12–75)	70 %
SCCO-HT (*n* = 18)	26 (8–40)	50 %
NET-O (*n* = 18)	62 (13–76)	33 %
SCLC (*n* = 58)	66 (36–86)	65 %

**Fig. 1:**
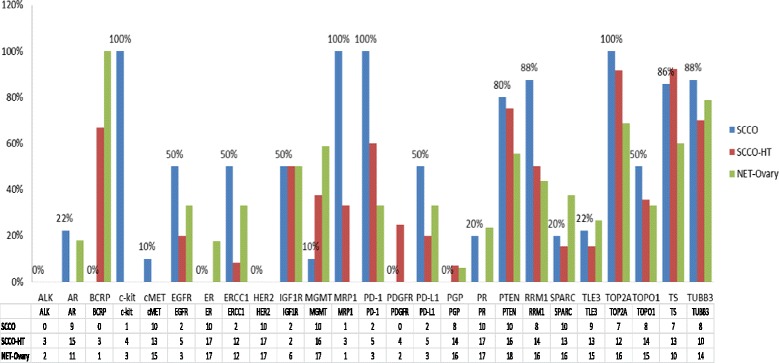
Biomarker Differences btw Rare Ovarian Cancers by IHC

By IHC, 100 % of SCCO patients (2/2), 60 % of SCCO-HT patients (3/5), and 33 % of NET-O (1/3) subtypes expressed PD1, while 42 % of SCLC patients (22/53) expressed PD1. Moreover, 50 % of SCCO patients (1/2), 20 % of SCCO-HT patients (1/5), and 33 % of NET-O patients (1/3) expressed PDL1, while 0 patients with SCLC (0/54) expressed PDL1. (SCCO 1/2 vs. SCLC 0/54 [*p*-value = 0.036]). In addition, by IHC, 100 % of SCCO patients (7/7), 92 % of SCCO-HT patients (11/12), and 69 % of NET-O patients (11/16) expressed TOP2A, while 95 % of patients with SCLC (52/55) expressed TOP2A. (NET-O 11/16 vs. SCLC 52/55 [*p*-value = 0.012]). Also 8 % (1/12) of SCCO-HT patients, 33 % (4/12) of NET-O, and 50 % (1/2) SCCO patients expressed ERCC1. Moreover, 10 % (1/10) of SCCO patients, 38 % (6/16) of SCCO-HT patients, and 59 % (10/17) of NET-O patients expressed MGMT, while 13 % of SCLC patients (7/55) expressed MGMT. (NET-O 10/17 vs. SCLC 7/55 [*p*-value =0.0003]). Also, 88 % (7/8) of SCCO patients expressed RRM1. SCCO and SCLC patients had similar patterns of other IHC expression (Fig. 1).

**Fig. 2:**
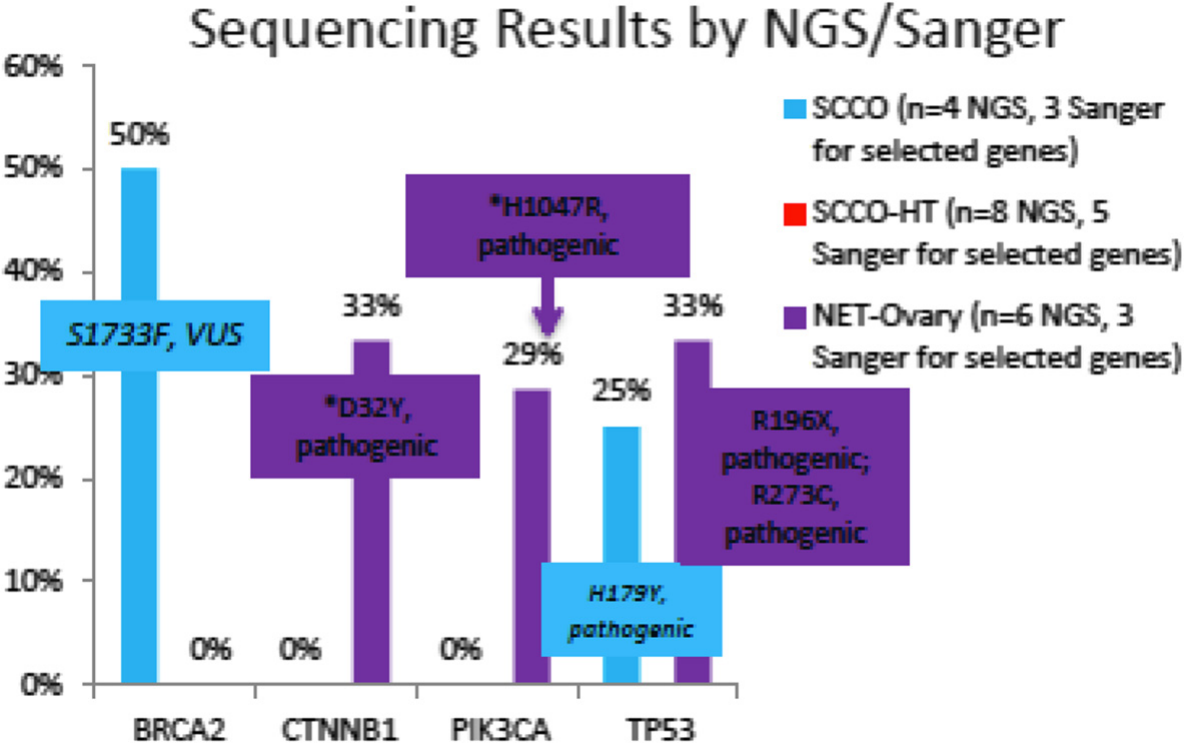
Sequencing results by NGS/Sanger

No amplifications were identified in cMET, EGFR, or HER2 by ISH in any neuroendocrine cancer of the ovary. PIK3CA was amplified by ISH in NET-O patients in 33 % (1/3) of patients (Table 2). NGS and Sanger sequencing of a 47-gene panel revealed TP53 mutations in 25 % of SCCO (1/4) patients and BRCA2 mutations in 50 % of SCCO (1/2) patients. PIK3CA mutations were seen in 16 % (1/6) of sequenced NET-O patients, but no SCLC patients. No other actionable mutations were identified (Fig. 2).

**Table 2:** Amplifications detected by in situ hybrization

	cMET	EGFR	HER2	PIK3CA
SCCO	0/4	0/2	0/9	n/a
SCCO-HT	0/9	0/2	0/13	n/a
NET-O	0/10	0/1	0/15	1/3

## Discussion

In contrast to other tumor types, Caris Molecular Intelligence did not identify recurrent actionable mutations or alterations in the subtypes of SCCO. IHC patterns suggested potential sensitivities to chemotherapy. Alternative methods of genomic interrogation, such as total exon sequencing, may discover actionable targets. Neuroendocrine cancers of the ovary were similar when profiled by Caris Molecular Intelligence to SCLC.

IHC testing for biomarker differences between these rare tumor subtypes showed high rates of TOP2A (100 % of SCCO patients, 92 % of SCCO-HT patients and 69 % of NET-O patients) in all 3 subtypes of neuroendocrine cancers of the ovary, implicating the use of topoisomerase inhibitors as rationale treatment options. Topoisomerase inhibitors such as pegylated liposomal doxorubicin have been commonly used as second line therapy in platinum resistant ovarian cancer [13]. Thibault et al. showed that inhibition of topoisomerase II using polyamine vectorized inhibitor (F14512) significantly inhibited tumor growth in ovarian cells and constitutes a potential new therapy for platinum resistant ovarian cancer [14].

Platinum-based therapies are the current standard of care for ovarian cancer. However, drug resistance plays an important role in determining overall survival. Expression of ERCC1, a strategic marker of nucleotide excision repair, has been associated with resistance to platinum based chemotherapy [15]. SCCO-HT exhibited the lowest rate of ERCC1 in our study, implicating the use of platinum based regimens for this sub-type.

MGMT, a DNA repair enzyme, plays a crucial role in mediating resistance to alkylating agents in various tumors, especially malignant gliomas. Epigenetic silencing of this gene has resulted in increased chemo-sensitivity by compromising DNA repair mechanisms. Temozolomide, an alkylating agent, is currently being tested as a single agent for ovarian cancer but has the potential for second line therapy in combination with methoxyamine for patients that have failed platinum and paclitaxel chemotherapy [16, 17]. Our data showed that SCCO exhibited the lowest rate of MGMT, implicating alkylating agents such as temozolomide as a potential agent to be used.

Elevated levels of RRM1 gene have been associated with poor outcomes in advanced NSCLC treated with gemcitabine [18, 19]. Several studies have been conducted to explore the potential benefit of gemcitabine in ovarian cancer. Ferrandina et al. looked at both RRM1 and RRM2 in primary ovarian cancer and observed shorter OS with higher RRM2 expression [20]. Our cases of SCCO had high expression of RRM1 but NET-O exhibited the lowest rate of RRM1 expression, implicating gemcitabine as a useful agent for NET-O.

Blocking the PD1/PDL1 interaction is being studied in several malignancies with good responses. Our subset of SCCO had a high expression of PD1 and PDL1 and the therapeutic potential of this pathway needs to be explored.

Mutations detected by hot spot sequencing panel were infrequent events and amplification by ISH were rare as well. Rare ovarian cancers would benefit from expanded mutation profiling to identify additional potential targets as well as confirm the presence of SMARCA4 in SCCO-HT.

Our study had some limitations. This was a retrospective analysis of a limited number of cases. Due to the rarity of this disease only 46 cases were identified and statistical analysis could not be performed. The lack of information such as patient demographics, treatment modalities and outcome prevented us from determining clinical correlation.

## Conclusion

Neuroendocrine cancers of the ovary are rare malignancies. With no recurrent actionable mutations, treatment remains a challenge. Future clinical trials looking at standard of care chemotherapy using platinum based chemotherapeutics and comparing that to specific chemotherapy regimens based off of IHC mutations may prove to be of significance in treating this rare disease. Our study showed that all SCCO types showed high expression of TOP2A, therefore, trials comparing platinum based therapies to topoisomerase II inhibitors such as doxorubicin or etoposide in patients who have high expression of ERCC1, which is associated with platinum resistance, may lead to a change in our approach to this subset of diseases. Also, there is potential for clinical trials comparing standard chemotherapy to PD1/PDL1 inhibition in SCCO as our subset of SCCO patients showed increased expression of PD1 and PDL1. Novel treatment options can be used on a case-by-case basis depending on the genomic profile. Prospective studies need to be conducted for better results, but given the rarity of this disease, that may prove to be difficult.

## Abbreviations

CISH: Chromogenic in situ hybridization
FISH: Fluorescence in situ hybridization
IHC: Immunohistochemistry
NET-O: Neuroendocrine tumor of the ovary
NGS: Next generation sequencing
NSCLC: Non-small cell lung carcinoma
SCCO: Small cell carcinoma of the ovary
SCCO-HT: Small cell carcinoma of the ovary–hypercalcemic type
SCLC: Small cell lung carcinoma
